# Subclinical signs of podocyte injury associated with Circulating Anodic Antigen (CAA) in *Schistosoma mansoni*-infected patients in Brazil

**DOI:** 10.1590/0037-8682-0341-2022

**Published:** 2023-02-20

**Authors:** Mariana Silva Sousa, Gdayllon Cavalcante Meneses, Govert Jan van Dam, Paul Leo Albert Maria Corstjens, Rosangela Lima de Freitas Galvão, Marta Cristhiany Cunha Pinheiro, Alice Maria Costa Martins, Elizabeth de Francesco Daher, Fernando Schemelzer de Moraes Bezerra

**Affiliations:** 1 Universidade Federal do Ceará, Departamento de Análises Clínicas e Toxicológicas, Laboratório de Pesquisa em Parasitologia e Biologia de Moluscos, Fortaleza, CE, Brasil.; 2 Universidade Federal do Ceará, Programa de Pós-graduação stricto senso em Ciências Médicas, Fortaleza, CE, Brasil.; 3 Leiden University Medical Centre, Department of Parasitology, Leiden, The Netherlands.; 4 Leiden University Medical Centre, Department of Cell and Chemical Biology, Leiden, The Netherlands.; 5 Universidade Federal do Ceará, Programa de Pós-graduação stricto senso em Patologia, Fortaleza, CE, Brasil.; 6 Universidade Federal do Ceará, Programa de Pós-graduação stricto senso em Ciências Farmacêuticas, Fortaleza, CE, Brasil.

**Keywords:** Schistosomiasis, Kidney disease, Up-Converting Phosphor Reporter Particle, Lateral Flow Circulating Anodic Antigen (UCP-LF CAA) assay, biomarkers, Vascular Endothelial Growth Factor (VEGF)

## Abstract

**Background:**

The long-term effects of schistosomiasis on the glomerulus may contribute to the development of chronic kidney disease. This study aimed to investigate baseline *Schistosoma mansoni*-Circulating Anodic Antigen (CAA) levels and their association with kidney biomarkers related to podocyte injury and inflammation in long-term follow-up after praziquantel (PZQ) treatment.

**Methods:**

*Schistosoma* infection was diagnosed by detecting CAA in urine using a quantitative assay based on lateral flow using luminescent up-converting phosphor reporter particles. A cutoff threshold of 0.1 pg/mL CAA was used to diagnose *Schistosoma* infection (baseline) in a low-prevalence area in Ceará, Northeast, Brazil. Two groups were included: CAA-positive and CAA-negative individuals, both of which received a single dose of PZQ at baseline. Urinary samples from 55 individuals were evaluated before (baseline) and at 1, 2, and 3 years after PZQ treatment. At all time points, kidney biomarkers were quantified in urine and adjusted for urinary creatinine levels.

**Results:**

CAA-positive patients had increased baseline albuminuria and proteinuria and showed greater associations between kidney biomarkers. CAA levels correlated only with Vascular Endothelial Growth Factor (VEGF) (podocyte injury) levels. Increasing trends were observed for malondialdehyde (oxidative stress), monocyte chemoattractant protein-1 (inflammation marker), and VEGF. In the follow-up analysis, no relevant differences were observed in kidney biomarkers between the groups and different periods.

**Conclusions:**

*S. mansoni*-infected individuals presented subclinical signs of glomerular damage that may reflect podocyte injury. However, no causal effect on long-term renal function was observed after PZQ treatment.

## INTRODUCTION

Despite the reduction in mortality and morbidity, schistosomiasis was reported in 8,756 deaths between 2000 and 2011 in Brazil and remains an important public health issue^1^. According to a national prevalence survey (2010-2015), an estimated 1.5 million people are infected with this disease in Brazil^2^. Severe clinical forms of schistosomiasis may be present even in low-endemic areas^3^, and a spatiotemporal analysis identified high-risk clusters of death, mainly in areas along the coast of Brazil's Northeast Region^4^.

Renal involvement in schistosomiasis mansoni is characterized by glomerular changes^5^ ^,^ ^6^, although renal tubular damage has been reported^7^. Recently, a case report described *S. mansoni* infection as a trigger for the development of collapsing glomerulopathy in a patient with a high-risk APOL1 genotype^8^.

Therapeutic interventions performed in endemic areas of Brazil do not seem to have reduced the prevalence of *S. mansoni* glomerulopathy, although credible and reliable evidence is lacking^9^ ^-^ ^10^. The incidence of renal involvement in schistosomiasis varies from 5% to 6% in patients with schistosomiasis, whereas it increases by up to 15% in the hepatosplenic form^11^.

The diagnosis of *S. mansoni* infections is routinely performed through microscopic detection of parasite eggs in stool^12^ using the Kato-Katz technique. However, this diagnostic technique has lower sensitivity in areas of low endemicity, which results in an underestimation of infection prevalence^13^. 

Assays for the detection of *Schistosoma* circulating antigens (gut-associated antigens of adult worm) have been extensively described and are considered promising. Circulating cathodic antigen (CCA) and circulating anodic antigen (CAA) are both used to diagnose ongoing worm infections^14^ ^,^ ^15^ ^,^ ^16^. The CAA assay applies sensitive and quantitative up-converting reporter particle (UCP) technology in combination with a user-friendly lateral flow test platform (UCP-LF CAA assay) and can be applied to serum and urine^14^.

The test has been successfully applied in low-transmission settings in the People’s Republic of China, Tanzania, and Burundi for the diagnosis of *S. japonicum* ^17^ *S. haematobium* ^18^ and *S. mansoni* infections, respectively^19^, and was applied in Brazil, as reported in an accompanying previous article^20^, which evaluated the performance of the UCP-LF CAA assay to determine *S. mansoni* infections in the same study area.

Schistosomal glomerulopathy is an example of immune complex-induced parasitic nephropathy^21^. Kidney biopsies from individuals with active *S. mansoni* showed deposits of circulating antigens (CAA and CCA). In renal glomeruli, these antigens are major contributors to the pathogenesis of schistosomal glomerulonephritis^22^.

New kidney biomarkers have been studied in different clinical contexts, showing greater specificity and sensitivity than classic clinical kidney markers^23^. Monocyte chemoattractant protein-1 (MCP-1) is one of the most widely studied biomarkers of glomerulopathies and is associated with glomerular inflammation and interstitial nephritis^24^. Studies have shown that MCP-1 plays a central role in tubulointerstitial and glomerular lesions in membranoproliferative glomerulonephritis^25^, lupus nephritis^26^, crescentic glomerulonephritis^27^, diabetic nephropathy^28^, and IgA nephropathy^29^. Elevated urinary MCP-1 levels have also been reported in patients with visceral leishmaniasis^30^. In the chronic intestinal form of schistosomiasis, high urinary MCP-1 levels showed subclinical glomerular kidney injury in *S. mansoni*-infected patients residing in an area of low endemicity^31^.

Another new biomarker is Vascular Endothelial Growth Factor (VEGF), which is essential for the maintenance of the glomerular filtration barrier^32^. The serum levels of VEGF are increased in patients with active lupus nephritis^33^, and its urinary levels reflect podocyte damage in diabetic nephropathy^34^. This growth factor plays an important role in the pathogenesis of several diseases, including cancer and coronavirus disease^35^. Regarding infectious and parasitic diseases, a recent study showed that patients infected with *S. mansoni* and without clinical kidney disease had significantly higher urinary VEGF levels than the schistosomiasis-negative group^36^.

The long-term impacts of the initial infection have not yet been investigated, and glomerular involvement may be critical for the development of chronic kidney disease (CKD). Hence, the present study aimed to evaluate the involvement of glomerular damage biomarkers in patients diagnosed with *S. mansoni* infection from an area of low endemicity in Brazil. Additionally, a long-term study was conducted to evaluate the causal effect of *S. mansoni* infection on renal function.

## METHODS

### Ethics, recruitment, and treatment

The study protocol was approved by the Federal University of Ceará (UFC) Ethical Committee (Opinion N. 3.706.472) and was conducted with adherence to the Resolution N. 466/12 of the Brazilian Health Council and to the Declaration of Helsinki, as revised in 1975, 1983, 1989, 1996, and 2000. 

Treatment with praziquantel (PZQ) (Farmanguinhos, Ministry of Health, Brazil) was offered to all individuals free of charge, regardless of infection status at baseline. It was performed with a single dose of 60 mg/kg for children (≤15 years old) and 50 mg/kg for adults, as recommended by the Brazilian Ministry of Health^37^.

### Study area and population

The study used a longitudinal design and was carried out in the community of Bananeiras, a rural locality that belongs to the Capistrano municipality in Ceará state, Northeast Brazil (geographical coordinates: 4º 28' 20” S latitude, 38º 54' 14” W longitude). The KK technique revealed only four positive stool samples (1.6%) in this community^20^. 

### Inclusion criteria

To be included, the individuals had to meet the following criteria at baseline: 1) age ≥ 15 years at recruitment; 2) informed consent; 3) no recent treatment for schistosomiasis (at least within the past two years); and 4) no kidney disease, diabetes, and/or hypertension. 

The study consisted of two groups based on the detection of *S. mansoni* CAA at baseline: a group of *S. mansoni*-infected individuals, CAA-positive (PG), and a group of individuals not infected by *S. mansoni*, CAA-negative (NG). All individuals in both groups received PZQ treatment and underwent long-term evaluation- one, two, and three years after treatment.

### Sample collection

At baseline, one day before the collection day, plastic containers labeled with specific identification numbers were delivered to each study participant. The following day, the community was invited to return the containers filled with a fresh morning urine sample to fieldworkers stationed at Bananeiras Health Center. Aliquots of urine (5 mL) were frozen and stored at -20°C at the Parasitology and Mollusks Biology Research Laboratory at UFC in Brazil, prior to their transfer to the Leiden University Medical Center (LUMC) in the Netherlands for CAA testing. Smaller aliquots (1mL) remained in Brazil for the measurement of urinary kidney biomarkers. Urine samples were collected again at one, two, and three years post-treatment using the same procedures. Only one urine sample was collected at each time point. 

### *S. mansoni* infection diagnosis by UCP-LF CAA assays at baseline

Baseline urine samples were frozen and transported on dry ice to LUMC, where they were stored at -20°C. Urine samples were analyzed using a highly sensitive concentration-based assay (UCAA2000 format of the UCP-LF CAA test)^14^. Briefly, 2 mL urine samples were diluted with an equal volume of 4% (w/v) trichloroacetic acid and centrifuged, and then the clear supernatants were reduced to 20-30 µL amounts using an Amicon Ultra-4 device (EMD Millipore; Billerica, MA, USA) with a 10 kDa molecular weight cutoff. After incubation with the UCP-antibody conjugate, LF was initiated^14^. Strips were scanned for bound UCP using a Packard FluoroCount microtiter plate reader adapted with an IR laser (980 nm) modified to scan LF strips^38^.

Moreover, standard curves of CAA spiked in negative urine samples were used to quantify CAA levels in the clinical samples^15^. The assay cutoff of 0.1 pg/mL was confirmed by Corstjens et al^39^.

### Urinary kidney biomarkers

Urinary creatinine and albumin levels were quantified by immunoturbidimetry (COBAS C111, Roche^®^). Proteinuria was quantified using a colorimetric method through a reaction with pyrogallol red (Labtest^®^ MG, Brazil). Urinary oxidative stress was assessed using urinary malondialdehyde (MDA) levels, which react with thiobarbituric acid. MCP-1/CCL2 and VEGF were quantified by ELISA according to the manufacturer’s standards (R&D Systems, Minneapolis, MN, USA).

The ASYS Expert Plus model was used for colorimetric reading based on the detection limits of the kits. The detection limits were 15.6 pg/mL and 31.3 pg/mL for MCP-1 and VEGF, respectively. 

### Statistical analysis

Statistical analyses were performed using SPSS software version 20 (IBM Corp., Armonk, USA). Descriptive statistics are expressed as means and standard deviations or medians with interquartile ranges for continuous variables and frequency counts (percentages) for categorical data. CAA and kidney biomarkers were expressed based on the urinary creatinine ratio^40^. Normal distribution was verified using the Kolmogorov-Smirnov test. Levene's test was used to compare variability between groups. Continuous variables were compared using Student's T or Mann-Whitney test. Paired analysis aimed at comparing biomarkers during the participants’ post-treatment follow-up was performed using Friedman’s test, followed by pairwise comparisons with Wilcoxon’s test. To avoid type I error, the critical *p* value was adjusted according to the number of groups: 0.05/4 = 0.0125. Thus, for pairwise comparisons, p ≤ 0.0125 among the groups was considered statistically significant. Categorical variables were compared using the chi-squared test. Spearman's rho coefficient was used to determine correlations between the analyzed variables. Univariate regression analysis was used to determine the association between kidney biomarkers and antigens and unfavorable renal outcomes. All tests were two-tailed, and a 5% level of significance was adopted for all inferential procedures.

## RESULTS

### Study group characteristics and adherence

A total of 55 patients were analyzed: 38 *S. mansoni*-infected patients (PG) and 17 uninfected patients (NG). The patients had no clinically relevant kidney diseases. The study included 27 men (49.1%). The median CAA level in the PG was 1.8 pg/mL (0.7-4.1) and 1.6 pg/mL (0.6-3.6) for uncorrected and corrected for urinary creatinine levels, respectively. The patients’ characteristics are shown in Table 1.

**TABLE 1:** Characteristics of CAA-positive and CAA-negative *S. mansoni* patient groups at baseline, Brazil^a^.

	PG	NG	
	(n=38)	(n=17)	p value
Male sex, nº (%)	17 (45)	10 (59)	0.334
Age	39.4 [15.7]	38.7 [12]	0.873
Glycemia, mg/dL	107.6 [22.3]	103.3 [12.6]	0.489
SBP, mmHg	12.9 [2]	12.4 [1.4]	0.399
DBP, mmHg	8.4 [1.4]	8.1 [1]	0.341
UCAA2000-,pg/mg-Cr^b^	1.6 (0.6 - 3.6)	-	

^a^Data are expressed as mean, with standard deviation in brackets or as median and interquartile range in parentheses except as indicated. Chi-square test was applied for categorical data; Student's t and Mann-Whitney tests were used for normally and non-normally distributed data, respectively. Urine CAA levels were corrected for urinary creatinine (Cr) levels. **CAA:** Circulating Anodic Antigen; **PG:** CAA-Positive Group; **NG:** CAA-Negative Group; **SBP:** Systolic Blood Pressure; **DBP:** Diastolic Blood Pressure. ^b^UCAA 2000-: UpConverting reporter Particle Lateral Flow Circulating Anodic Antigen (UCP-LF CAA) assay prepared with 2 mL of urine, indecisive results were considered negative.

### Renal parameters at baseline

There was a significant increase in albuminuria and proteinuria in the PG group before treatment, as shown in Table 2.

**FIGURE 1:**
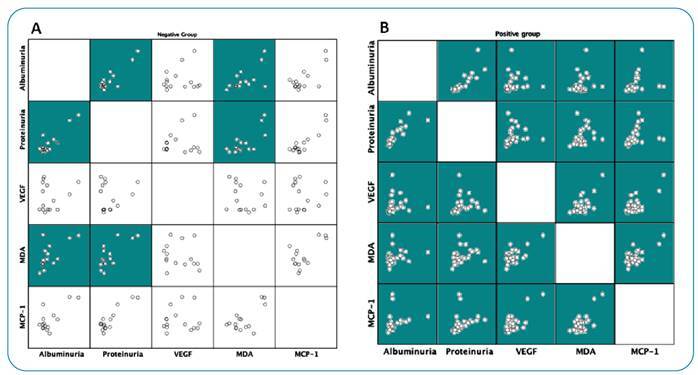
Matrix scatter plots of the baseline renal parameters in CAA-negative and CAA-positive groups. The green squares represent a significant correlation (p <0.05). **VEGF:** Vascular Endothelial Growth Factor; **MDA:** Malondialdehyde; **MCP-1:** Monocyte Chemoattractant Protein-1.

Increasing trends were observed for MDA, MCP-1, and VEGF, but these were not statistically significant (Table 2). Nonetheless, by performing a correlation analysis within each group, significant associations between the biomarkers were observed in PG (Figure 1).

**TABLE 2:** Renal parameters of CAA-positive and CAA-negative *S. mansoni* patient groups at baseline, Brazil^a^.

	PG (no. patients =38)	NG (no. patients =17)	p value
Albuminuria, mg/g-Cr	3.82 (2.18 - 7.58)	1.74 (1.25 - 2.77)	0.005
Proteinuria, mg/g-Cr	79.28 (64.73 - 119.70)	57.68 (53.58 - 75.93)	0.025
VEGF, pg/mg-Cr	31.19 (14.38 - 50.04)	23.78 (9.55 - 53.41)	0.346
MDA, µmol/mg-Cr	5.89 (5.08 - 6.97)	5.12 (4.56 - 6.04)	0.103
MCP-1, pg/mg-Cr	82.70 (58.01 - 127.97)	63.11 (52.79 - 93.32)	0.171

^a^Data are expressed as median and interquartile range in parentheses. Mann-Whitney test was used for non-normally distributed data. All kidney biomarkers were corrected for urinary creatinine (Cr) levels. **CAA:** Circulating Anodic Antigen; **PG:** CAA-Positive Group; **NG:** CAA-Negative Group; **VEGF:** Vascular Endothelial Growth Factor; **MDA:** Malonaldehyde; **MCP-1:** Monocyte Chemoattractant Protein-1.

### Correlation of *S. mansoni* urine CAA levels with renal parameters at baseline

CAA levels correlated only with VEGF levels, showing an association at baseline between antigen concentration and podocyte injury biomarkers (Table 3). However, the correlation coefficient was 0.43, which represents a low association, and its clinical relevance should be evaluated.

**TABLE 3:** Correlation of urine CAA with renal parameters in the PG at baseline, Brazil^a^.

	UCAA2000-, pg/mg-Cr^b^	
PG (no. patients =38)	Rho	p value
Albuminuria, mg/g-Cr	0.042	0.809
Proteinuria, mg/g-Cr	0.009	0.959
VEGF, pg/mg-Cr	0.425	0.012
MDA, umol/g-Cr	0.149	0.393
MCP-1, pg/mg-Cr	-0.010	0.954

^a^Spearman’s correlation analysis; Rho coefficient. CAA and kidney biomarkers were corrected for urinary creatinine (Cr) levels. **CAA:** Circulating Anodic Antigen; **PG:** CAA-Positive Group; **VEGF:** Vascular Endothelial Growth Factor; **MDA:** Malonaldehyde; **MCP-1:** Monocyte Chemoattractant Protein-1. ^b^UCAA 2000-: UpConverting reporter Particle Lateral Flow Circulating Anodic Antigen (UCP-LF CAA) assay prepared with 2 mL of urine, indecisive results were considered negative.

### Post-treatment follow-up analysis

**FIGURE 2:**
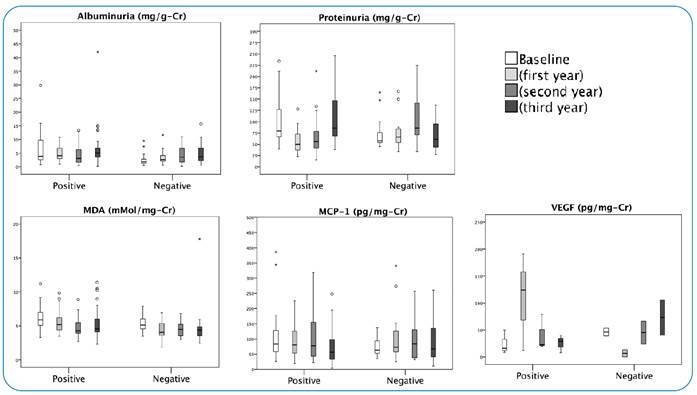
The levels of renal parameters at post-treatment prospective follow-up in CAA-positive and CAA-negative groups. The four cross-sectional study time points are shown: baseline (T = 0) and 1, 2, and 3 years after treatment. Albuminuria; **MDA:** Malonaldehyde; **MCP-1:** Monocyte Chemoattractant Protein-1, Proteinuria; **VEGF:** Vascular Endothelial Growth Factor.

Figure 2 shows the concentrations of each kidney biomarker at the four cross-sectional time points in PG and NG. In relation to the NG group, no statistical significance was observed for all biomarkers, except for urinary MDA regarding comparison between “baseline” and “Second year” (p=0.009).

The PG group showed no statistically significant differences in albuminuria (p=0.098), MCP-1 (p=0.139), and VEGF (p=0.457) biomarkers. However, regarding proteinuria, statistical significance was detected for “baseline” vs “First year”; “baseline” vs “Second year”; “First year” vs “Third year”; and “Second year” vs “Third year” (p<0.001). After baseline, proteinuria decreased in the first and second years and increased again in the third year of follow-up.

Moreover, for urinary MDA, “baseline” vs “Second year” and “First year” vs “second year” also showed significant differences (p<0.001). Similar to proteinuria, MDA levels decreased in the two years following the baseline.

Due to the detection of associations between biomarkers in the PG at baseline (Figure 1), correlation analyses of baseline VEGF and CAA with renal outcome in the post-treatment prospective follow-up were performed in this group.

An increase in albuminuria at 1, 2, or 3 years after treatment was considered an unfavorable outcome, since increased albuminuria is, according to the Kidney Disease Improving Global Outcomes (KDIGO) criteria, a diagnostic parameter for CKD. Nevertheless, baseline VEGF and CAA levels for PG were not predictors of albuminuria increase at 1, 2, or 3 years post-treatment in the Spearman’s correlation analysis. Similar negative results were obtained on linear regression analyses for both markers.

## DISCUSSION

This was the first study to investigate the association between *S. mansoni* infection based on the CAA assay and biomarkers of renal injury, and to show a correlation between urine CAA levels and urinary VEGF levels, a biomarker associated with podocyte injury. 

CAA and CCA are the main antigens implicated in schistosomal glomerulonephritis pathogenesis. In this study, the patients were asymptomatic, although they showed increased signs of glomerular damage, corroborating the findings of other *S. mansoni* experimental and clinical studies in which renal injury was attributed to mechanisms that lead to glomerular alterations^22^ ^,^ ^41^ ^,^ ^42^.

In the current study, the increased signs of glomerular damage observed in *S. mansoni*-infected patients were demonstrated before treatment and thus were due to the parasitic infection itself. In fact, schistosomiasis glomerular damage is characterized by tissue damage from the deposition of immune complexes of parasite-circulating antigens, which may lead to proliferative glomerulonephritis^43^.

In the present study, comparison of general characteristics did not identify any differences between the CAA-positive and CAA-negative groups. However, a correlation between the urinary levels of CAA and VEGF was also observed. In a recent cross-sectional study that investigated the association between parasite loads of *S. mansoni* and biomarkers of kidney injury, patients residing in areas of high endemicity for schistosomiasis mansoni, diagnosed using the Kato-Katz technique, presented with urinary albumin levels within the normal range. However, urinary VEGF levels were significantly higher than those in the control group^36^. 

The difficulty in diagnosing *S. mansoni* infection using the Kato-Katz technique due to its low sensitivity in areas of low endemicity has been previously demonstrated in the literature^13^ and equally verified in previous articles that evaluated the performance of the POC-CCA test ^44^ and the UCP-LF CAA assay^20^. The latter assay was used to detect and quantify CAA as a diagnostic tool for the stratification of this study’s analysis groups. Unfortunately, it would not be possible to stratify the groups using the Kato-Katz technique, as only four patients were diagnosed using this approach in the assessed community, as described previously^20^. Subsequent studies in moderate to high endemicity areas for schistosomiasis, where the individuals have a higher worm load (with consequently higher CAA concentrations) and the parasitological technique is satisfactory, are needed to better elucidate the findings of this study. Investigations of parasitic load and its relationship with glomerular injury are important. A study carried out using an experimental model of *S. mansoni* reported a significant correlation between kidney damage and parasite burden^45^. However, in their study among residents of a high endemicity area, Galvão et al.^36^ demonstrated that renal damage seems to occur regardless of the parasitic load of *S. mansoni*. 

During the two years following baseline, the PG group showed a significant decrease in proteinuria and urinary MDA. Proteinuria levels increased again in the third year. Moreover, when a correlation analysis was performed within each group (CAA-positive and CAA-negative), greater associations were observed between the biomarkers in the PG, indicating that possible glomerular alterations may have occurred in the PG, reflecting the aforementioned increase in albuminuria and proteinuria. The presence of proteinuria in these patients is an important factor, and when elevated, it can accelerate the progression of renal disease through the induction of chemokines and activation of the complement system, which leads to infiltration of inflammatory cells into the renal interstitium^46^. However, in the present study, none of the patients had proteinuria at nephrotic levels, suggesting an insult at baseline, which explains the higher levels of proteinuria in comparison with the subsequent two years. In contrast to visceral leishmaniasis patients, in which elevated proteinuria may result from the presence of hypergammaglobulinemia^47^, a mechanism hypothesis here would be the presence of podocyte injury, with consequent glomerular filtration process impairment. The correlation between CAA and VEGF at baseline in this study suggests an association between the levels of this antigen responsible for schistosomiasis-associated kidney injury and the podocyte injury biomarker, which may aid in explaining the mechanism of kidney pathogenesis in these patients. 

Podocyte injury occurs through the reduction of its primary and secondary processes, causing a rupture in the barrier, resulting in the passage of molecules of clinical importance, such as albumin, and consequently, the appearance of these molecules in the urine. Therefore, podocyte loss cannot be compensated by the remaining healthy cells^48^. Urinary VEGF is an important factor in podocyte survival and is responsible for maintaining the integrity of the glomerular filtration barrier.

Excess or decreased levels of urinary VEGF can alter the development or maturation of podocytes, causing defects in their integrity. The decrease in its cytoplasmic processes and the deregulation of nephrin, an important protein for the maintenance of the cytoplasmic extension structures of podocytes, results in changes in the glomerulus and disruption of its filtration barrier^32^ ^,^ ^49^. In fact, other studies have shown that urinary VEGF reflects podocyte damage^34^ ^,^ ^50^. 

It is possible that the urine sample concentration step applied in the UCP-LF CAA assay was critical for the association of CAA with VEGF, as a large amount of CAA is retained in the glomerulus^5^ ^,^ ^41^ ^,^ ^42^. However, we believe that the deposition of immune complexes containing CAA in the kidney could also mediate the leakage of CAA into the urine, but it is difficult to establish a direct relationship. Moreover, little is known about the humoral antibodies against CAA, which may be absent. The pathophysiological mechanisms are still poorly understood in schistosomal glomerulopathy^21^. 

Clinical evidence and experimental models have demonstrated that MCP-1 plays a critical role in the development of kidney disease^51^. It plays a central role in membranoproliferative glomerulonephritis^52^, lupus nephritis^53^, crescentic glomerulonephritis^27^, diabetic nephropathy^28^, and immunoglobulin IgA nephropathy^29^. Studies on intestinal^31^ and hepatosplenic^7^ schistosomiasis, visceral leishmaniasis^54^, and leprosy^55^ have shown higher urinary MCP-1 levels, including urinary oxidative stress. In another study among patients infected with *S. mansoni* living in a high endemicity area, the median levels of urinary MCP-1 were higher than those of the control group, with no statistically significant difference^36^. In contrast, in the present study, PG only showed increasing trends in MCP-1 levels. However, MCP-1 and VEGF in the PG correlated with traditionally investigated kidney markers, corroborating the findings of Hanemann et al.^31^, who observed a correlation between urinary MCP-1 and albuminuria levels in a study of *S. mansoni*-infected patients. This was also observed in visceral leishmaniasis patients by Oliveira et al^30^, who found that the correlation among albuminuria, elevated urinary MCP-1 levels, and inflammation could represent the presence of macrophages in renal tissues. Similarly, Bezerra et al.^44^ reported a correlation between urinary MCP-1 levels and creatinine, urea, and albuminuria and an inverse correlation with glomerular filtration rate during hospital admission. Moreover, urinary MCP-1 is associated with increased albuminuria in kidney diseases, such as diabetic nephropathy^55^.

Of note, other factors, in addition to worm antigens, seem to contribute to the genesis of glomerular disease in schistosomiasis. Experimental studies have shown that portal vein clamping in rats favors immune complex deposits in the kidneys^56^. Liver disease impacts kidney damage^45^. Portal hypertension with collateral circulation and liver damage with an inefficient macrophage system seen in patients with the hepatosplenic form of the disease allows schistosomal antigens to escape hepatic clearance and bind to antibodies in the liver circulation and subsequently deposit in the glomeruli^57^ ^-^ ^59^. This explains the higher prevalence of glomerulopathy in the hepatosplenic form, although renal involvement may also be observed in the hepatointestinal form^31^. 

A biomarker panel assessment in the same clinical context is important, as it can complement each other's pathophysiological mechanisms and improve not only the understanding of nephropathy but also its clinical diagnosis^60^.

Although CAA was associated with VEGF and CAA-based stratification showed differences in albuminuria at baseline, PG showed no association with kidney injury progression in the long term. One hypothesis is that the extremely low parasitic load observed may not have decisively affected the renal tissue in any patient. Another issue is the possibility of treatment failure with PZQ^61^, resulting in the persistence of some CAA-positive patients even after treatment, although at a very low load. Another point to be considered is that parasitic treatment could possibly protect against further kidney injury and kidney disease progression. A limitation of our study is that blood VEGF was not measured to rule out the possibility that urinary VEGF could be of systemic origin. Another limitation was the small sample size of the analyzed participants. In addition, CAA levels were only collected at baseline. Thus, further prospective studies with good protocols are needed to elucidate the long-term renal impacts.

In summary, the observed correlation between urinary CAA and uVEGF levels may reflect podocyte injury, specifying the mechanisms of kidney injury and dysfunction in these patients. New kidney biomarkers that can detect subclinical alterations through non-invasive urinary examinations may be useful for the early diagnosis of renal involvement in schistosomiasis and for the prevention of renal disease progression in asymptomatic individuals.
